# Effects of gastric bypass on the digestibility and postprandial metabolic fate of ^15^N dietary protein in rats

**DOI:** 10.1371/journal.pone.0307075

**Published:** 2024-08-05

**Authors:** Soukaïna Benhaddou, Lara Ribeiro-Parenti, Nadezda Khodorova, Alexandra Willemetz, Martin Chapelais, Dalila Azzout-Marniche, Maude Le Gall, Claire Gaudichon

**Affiliations:** 1 AgroParisTech, INRAE, UMR PNCA, Université Paris-Saclay, Palaiseau, France; 2 Inserm UMRS 1149, Centre de Recherche sur l’Inflammation, Université Paris Cité, Paris, France; 3 Service de Chirurgie Digestive Oesogastrique et Bariatrique, Hôpital Bichat—Claude‐Bernard, Assistance Publique‐Hôpitaux de Paris, Université Paris Cité, Paris, France; National Healthcare Group, SINGAPORE

## Abstract

Roux-en-Y Gastric Bypass may be associated with an alteration of protein bioavailability in relation to intestinal remodeling. Our study aimed to test this hypothesis by Roux-en-Y Gastric Bypass. Diet-induced obese rats underwent Roux-en-Y Gastric Bypass surgery (RYGB rats) while a Sham-operated control group was used. All rats received a ^15^N-labeled protein meal 1 or 3 months after surgery and were euthanized 6h later. Protein digestibility, ^15^N recovered in organs and urea pool, fractional protein synthesis rate, and intestinal morphometry were assessed. Protein digestibility was similar in all groups (94.2±0.3%). The small intestine was hypertrophied in RYGB rats 1 month after surgery, weighing 9.1±0.2g vs. 7.0±0.3g in Sham rats (P = 0.003). Villus height and crypt depth were increased in the alimentary limb and ileum of RYGB rats. However, Roux-en-Y Gastric Bypass had no impact on the fractional synthesis rate. In the gastrointestinal tract, ^15^N retention only differed in the ileal mucosa and was higher in RYGB rats at 1 month (0.48±0.2% vs. 0.3±0.09%, P = 0.03). ^15^N recovery from the liver, muscle, and skin was lower in RYGB rats at 1 month. ^15^N recovery from urinary and plasma urea was higher in RYGB rats at both times, resulting in increased total deamination (13.2±0.9% vs. 10.1±0.5%, P<0.01). This study showed that Roux-en-Y Gastric Bypass did not affect protein digestibility. Dietary nitrogen sequestration was transitorily and moderately diminished in several organs. This was associated with a sustained elevation of postprandial deamination after Roux-en-Y Gastric Bypass, whose mechanisms merit further studies.

## Introduction

Bariatric surgery is one of the most effective treatments for adults with severe obesity and its comorbidities. In particular, Roux-en-Y-Gastric Bypass (RYGB) is a procedure that reduces the size of the stomach and bypasses the duodenum as well as the first 40 to 50 cm of jejunum named the biliopancreatic limb. The new gastric pouch is directly connected to a roux limb, typically 75 to 150 cm. Most micro- and macro-nutrients will thus be absorbed in the common limb, which is distal to where the biliopancreatic and the roux limb connect [1]. RYGB produces many beneficial effects, such as sustained weight loss and improved comorbidities, not only because of the reduction in food intake and malabsorption but also because of multiple hormonal and neuronal mechanisms [2]. However, insufficient weight loss or weight regain may occur after this surgery [3], and complications can also result from RYGB, such as nutritional deficiencies in vitamins (B1, B12, A, D, E, K), iron and protein [4]. However, the effect of gastric bypass on protein deficiency has been minimally explored. The scarcity of studies primarily stems from the lack of sensitive markers for early protein status degradation, with biological or clinical signs only manifesting late. Although plasma albumin (levels <35g/L) or pre-albumin (<110mg/L) are widely employed, they mostly reveal severe protein malnutrition [5]. The prevalence of hypoalbuminemia is variable in the literature. For instance, a low prevalence ranging from 1.3% to 4.7%, was reported in some studies [6, 7]. Another study demonstrated a slight but significant decrease in plasma pre-albumin concentration at 1 month and 3 months post-RYGB in 57% of patients, whereas serum albumin levels remained unchanged [8]. The decline in pre-albumin within the first 6 months post-operation was confirmed in another study, which nevertheless showed that one year after bypass, pre-albumin levels returned to baseline [9]. A subsequent study evaluated pre-albumin levels at longer intervals, 1 year and 3 years after gastric bypass, indicating that pre-albumin levels were similar to those observed before surgery [10]. These studies suggest a transient disruption in protein status following gastric bypass. Moreover, lean mass is frequently affected with a loss varying from 10 to 36% depending on the study [11–14]. The mechanisms underlying the alteration of protein status after RYGB are poorly studied, and the obtained data are disparate and thus remain unclear. It has been supposed to result from the protein malabsorption induced by bypassing a portion of the small intestine in RYGB. Protein malabsorption may be negatively associated with the length of the common limb, especially in patients in whom it is too short [15]. Among nine patients with long-limb RYGB, Odstrcil *et al*. [16] found a decreased protein absorption coefficient in only four patients five months after surgery. Another study using a single test meal containing egg proteins labeled with stable isotopes indicated that protein digestion decreased nine months after RYGB [17]. Paradoxically, other studies using a test meal containing ^13^C-labeled caseinate revealed accelerated dietary AA absorption after RYGB in humans [18, 19].

In a previous rat study using a ^15^N meal test (containing milk proteins) [20], we observed a slight but significant increase in protein digestibility and intestinal mucosal hypertrophy induced by RYGB, which has also been reported by others [21–23]. Further, this study suggested a loss of the metabolic availability of dietary AAs (AAs), revealed by lower dietary nitrogen (N) recovery in the organs except for intestinal tissue [20]. Based on these observations, we hypothesized that RYGB increased the sequestration of dietary N in the hypertrophied intestinal mucosa at the expense of other organs.

The present study evaluated the time course adaptation to RYGB of the intestinal mucosa and the postprandial handling of dietary proteins in rats using a test meal containing ^15^N-labeled casein. We compared adaptation one month versus three months after gastric bypass. To our knowledge, data on the time course adaptation of dietary protein metabolism, particularly deamination, are lacking.

## Materials & methods

### Animals and diets

This study was carried out in compliance with European Union Directive 2010/63/EU for animal experiments and validated by the Institutional Animal Care and Use Committee (Comité d’Ethique Paris Nord, n°121, APAFIS #28833).

Forty-one diet-induced obese Male Wistar rats were purchased from Envigo (Gannat, France) and weighed 400-500g. They were fed a High-Fat Diet (HFD, composition in S1 Table) by the purchaser 16 weeks before their arrival. Once housed in our animal facility, they were kept under standard environmental conditions for two weeks before surgery, at a maintained temperature of 21°C-22°C, a 12/12h light/dark cycle, and with water and food (HFD) available *ad libitum*.

### Surgical procedures

The animals were 24 weeks old when they were randomly divided into two groups: RYGB (n = 25) and sham-operated (Sham, n = 16) [24]. These two groups were then separated into two further subgroups: followed after surgery for 1 month (RYGB: n = 12, Sham: n = 8) or 3 months (RYGB: n = 13, Sham: n = 8). One month post-gastric bypass in rats is equivalent to approximately one and a half years in humans, while 3 months post-operation in rats corresponds to five years in humans. This allows us to investigate the short and medium-term effects of gastric bypass surgery. The rats were fasted overnight before the operation. They were anesthetized by the gaseous inhalation of isoflurane (Vertflurane, Virbac, France). The procedures were performed as previously described [20].

### Roux-en-Y Gastric Bypass (RYGB)

The surgical procedure has been validated previously [20, 21, 23, 25, 26]. Briefly, it started with a laparotomy, followed by isolation of the stomach outside the abdominal cavity and removal of the non-glandular part of the stomach (forestomach) by the application of a staple line. A gastric pouch was created using another staple line parallel to the first one. The remaining gastric pouch corresponded to 20% of the initial stomach size. The jejunum was transected 20cm after the pylorus to create the alimentary limb, which was anastomosed to the gastric pouch, and the biliopancreatic limb was anastomosed 15cm distally to the gastrojejunal anastomosis. The average total length of the common limb was 70-80cm. The lengths of the alimentary and biliopancreatic limbs were chosen according to the initial description of the RYGB procedures in humans, with the same ratio translated to the rat. The survival rate was 64% (16/25).

### Sham

To mimic surgery, an unarmed staple gun was used to pinch the stomach and the jejunum was transected and immediately repaired. The survival rate was 100% (16/16).

Following all procedures, the laparotomy was closed using 4–0 vicryl and 3–0 vicryl (Ethicon) sutures to sew up the abdominal wall and the skin, respectively. An analgesic (Xylocaine, Astra, 10 mg/kg) was infiltrated along all the sutures to reduce pain, and an intramuscular injection of antibiotic (Penicillin, 20,000 U/kg, PanPharma, Boulogne Billancourt, France)) was administered.

### Postsurgical care

After surgery, the animals were placed in individual cages with a reversed light/dark cycle (dark period from 8:00 am to 8:00 pm) implemented one month before sacrifice. For the first two days after surgery, the rats were injected subcutaneously with 13ml/day of an isotonic polyionic solution (Bionolyte G5, Baxter, Guyancourt, France). Between days 3 and 5, the rats were refed with an HFD, which was soft and easy to ingest. Thereafter, they received a hydrated standard diet *at libitum* (see composition in S2 Table). Each animal was followed daily with respect to food intake, weight loss, health (lacrimal and nasal secretions, hydration, stool pattern), and behavior (grooming, mobility, stress, aggression) for two weeks after surgery. Sham animals received the same postoperative care and were pair-fed with operated animals once the dietary intake had stabilized.

### Habituation to the consumption of a single test meal

Ten days before the postprandial test and cull, the rats were accustomed to rapidly eating an entire meal comprising 4g (dry weight) of the semi-liquid standard diet between 8:00 am and 8:45 am, with free access to the diet between 11:00 am and 7:00 pm. Between these two periods, the rats were fasted but continued to have free access to water.

**Fig 1:**
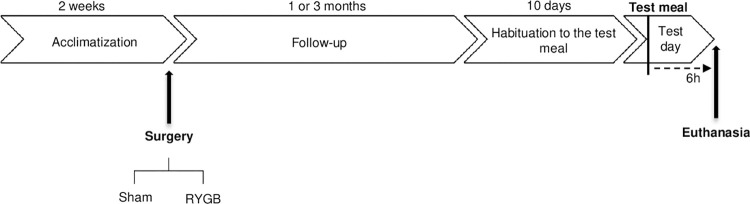
Design of the experimental procedure.

On the day of euthanasia, the rats were fed a test meal with the same composition as the standard diet but in which the protein (casein) was intrinsically labeled with ^15^N, as described previously [27]. Both groups of rats ingested the entire test meal within one hour. Fifteen minutes before euthanasia, the rats were injected in the lateral tail vein with a flooding dose (150μmol/100g of body weight) of [1-^13^C] valine (Euristop, Saint Aubin, France) under gaseous anesthesia. The rats were euthanized 6h after the meal by the gaseous inhalation of isoflurane overdose and then exsanguinated (Fig 1). The blood was collected, placed for 15 minutes at 4°C, and centrifuged, and the plasma was stored at -20°C. Different intestinal segments were identified depending on the surgical procedure and collected: stomach, jejunum (for Sham rats) or alimentary, biliopancreatic, and common limbs (for RYGB rats), ileum, cecum, and colon. The ileum was defined as the last 10cm of the small intestine, even if the ileum might be shorter [28], in order to obtain sufficient tissue and digesta for the analyses. The luminal content of the segments was rinsed with NaCl solution (9g/1Ll), and the entire contents were weighed and frozen at -20°C before being freeze-dried. Samples of each intestinal wall segment, liver, kidney, spleen, gastrocnemius muscle, and skin were collected, weighed, quickly frozen in liquid N, and stored at -20°C. They were then crushed under liquid N and freeze-dried. Other samples of intestinal segments were fixed for 24h in formalin and embedded in paraffin.

### Analytical methods

^15^N recovery in the digestive contents, organs, and urines was followed to assess the digestive and metabolic bioavailability of dietary N. Digestive and metabolic bioavailability was determined using an elemental analyzer (Vario Micro Cube, Elementar, Lyon, France) coupled with isotopic ratio mass spectrometry (Isoprime, GV Instrument, Manchester, UK). Atropine (Thermo Electron, Milano, Italy) was used as an elemental standard, and L-glutamic acid (USGS41, Sigma-Aldrich, St. Louis, MO) as an isotopic standard.

Individual AA digestibility was assessed by analyzing the AA composition in the meal and digestive contents of the cecum using an Ultra High-Performance Liquid Chromatography system (UHPLC, Acquity H-class, Waters) and by measuring the ^15^N-enrichment of AAs with Gas Chromatography-Combustion-Isotopic Ratio Mass Spectrometry (GC-C-IRMS, Isoprime, GV Instruments). The AA contents of the meal and digesta were determined after hydrolysis for 24h with HCl 6N at 110°C, with norvaline as an internal standard. AAs from samples and standards were derivatized using the AccQTag Ultra Derivatization Kit (Waters) and analyzed by UHPLC, as previously described [20, 29, 30]. For ^15^N enrichment in AAs, the hydrolysates were transferred to a hydrogen-form resin (Dowex 50WX8 hydrogen form 100–200 mesh; SigmaAldrich) and rinsed with NH_4_OH. AAs were derivatized with ethyl chloroformate, as previously described [31].

Plasma separation of the N fraction (proteins, free AAs, and urea) was achieved by first performing protein precipitation with 5-sulfosalicylic acid (100%). The protein pellet was then freeze-dried. The pH of the supernatant containing urea and free AAs was adjusted to 7 and transferred to a sodium-form resin (Dowex AG-50W AX8 100–200 mesh, Sigma-Aldrich). After incubation with urease at 30°C, the supernatant containing the free AAs was collected. The urea retained on the resin (in an ammonia form) was eluted with KHSO_4_.

Total urea in urine and plasma was determined using a biochemical assay assessed by a biochemistry platform (CRI, CEFI, Paris, France).

The *in vivo* protein synthesis rate in intestinal tissues was evaluated using the [^13^C]-valine flooding dose method, as described previously [32, 33]. The protein-bound fraction was hydrolyzed and derivatized with ethyl chloroformate, and GC-C-IRMS was used to quantify [^13^C]-valine enrichment in the fractions (Isoprime, GV Instrument, Manchester). Free AAs were isolated from the supernatant using a hydrogen-form resin and derivatized with N-tert-butyldimethylsilyl-N-methyltrifluoroacetamide mixed with 1% tert-butyldimethylchlorosilane and acetonitrile (Sigma-Aldrich). [^13^C]-valine enrichment was measured by Gas Chromatography (GC 6890N, Agilent Technologies, Les Ulis, France) coupled to Mass Spectrometry (MS 5973N, Agilent Technologies) (GC-MS) with electron impact ionization and selected ion monitoring (ion mass-to-charge ratio: 288 and 289).

### Histology

Three-micrometer slides were cut from each paraffin block to perform Hematoxylin Phloxine Saffron and Periodic Acid Shiff staining. Each slide was scanned using a Hamamatsu NanoZoomer (Tokyo, Japan), under NDP.view2 software. Villus height, crypt depth and serosa, muscularis propria, and submucosa height were all measured.

### Calculations

The recovery of dietary N in each tissue and the digestive contents was evaluated as

$$N_{exo}=N_{tot}\times \frac{APEsample}{APEmeal},$$

where N_exo_ is exogenous N (mmol), N_tot_ is the amount of N in the sample (mmol), and APE is the ^15^N-enrichment excess in the sample or the meal. APE represents the enrichment of the sample in atom percent (AP) minus natural enrichment. Natural enrichment values for the digesta [31] and organs [34] were obtained from previous studies of rats fed a milk protein diet. The estimation of dietary N in plasma protein was related to the plasma volume in the rat (3.5% of body weight) [35].

AA and peptide absorption were mostly determined in the duodenum and jejunum [36]. Thus, dietary N losses were measured in the distal parts of the intestine contents (ileum, cecum, colon, and feces). Orofecal digestibility (% of N ingested) was calculated as

$$Orofecal\;digestibility=\frac{Ning-(Nexo\;ileum+N\;exo\;cecum+N\;exo\;fecal)}{Ning}\times 100,$$

where N_ing_ is the amount of dietary N ingested (mmol), as previously described.

The orocaecal digestibility (%) of each AA was calculated as

$$AA\;orocaecal\;digestibility=\frac{AAing-AAexo\;cecum}{AAing}\times 100,$$

where AA_ing_ represents the total amount of ingested AA (mmol) and AA_exo cecum_ is the amount of dietary AAs (mmol) recovered in the contents of the cecum.

The recovery of dietary N in each tissue was evaluated as

$$N_{exo}=N_{tot}\times \frac{APEtissue}{APEmeal},$$

where N_exo_ is the exogenous AAs (mmol), N_tot_ is the amount of N in the tissue (mmol), and APE is the enrichment excess in the sample or the meal.

Dietary N contained in the urea body pool (mmol) was estimated by supposing that urea was uniformly distributed throughout total body water and calculated as

$$N_{diet}\;body\;urea=C_{plasma\;urea}\times 2\times \frac{0.67}{0.92}\times BW\times \frac{APEurea}{APEmeal},$$

where C_plasma urea_ is the concentration of urea in plasma (mmol/L), BW is the body weight of the rat (kg), APE_urea_ is the ^15^N-enrichment excess of urea in plasma samples, and APE_meal_ is the ^15^N-enrichment excess of the meal. In rats, the percentage of body water is assumed to be 67% of body weight, and water is 92% of plasma [37].

The fractional protein synthesis rate (FSR, in %/day) in each intestinal segment was calculated as

$$FSR=\frac{Eprotein-bound\;valine-Ebasal}{Efree\;valine\times Tinc}\times 24\times 60\times 100,$$

where E_free_ valine and E_protein-bound_ are the [^13^C] valine enrichment in free and protein-bound AAs, respectively. E_basal_ is the value of the leucine in the sample as a surrogate for basal valine enrichment [38]. The leucine and valine enrichment values measured by GC-c-IRMS were corrected for the number of carbon atoms added by derivatization. T_inc_ is the period for the incorporation of [^13^C] valine (in min) between the injection and euthanasia (15 minutes on average). 24 (h) and 60 (min) were used as factors to calculate the value as a percentage per day [39].

### Statistical analyses

All results are expressed as means ± sem. Follow-up data were analyzed using a mixed model for repeated measures with the group as a fixed factor and time as a repeated factor, with RStudio (RStudio Team (2020). RStudio: Integrated Development for R. RStudio, PBC, Boston). Otherwise, the differences between groups were tested using a two-way ANOVA with group and time (1 vs. 3 months) as factors and post hoc Bonferroni tests for pairwise comparisons. Differences between the villus height and crypt depth of the Sham jejunum and RYGB intestinal limbs were estimated using an unpaired Student’s t-test after verification that the normal distribution of data was not rejected. Differences were considered statistically significant at a value of P<0.05.

## Results

### Follow-up

**Fig 2:**
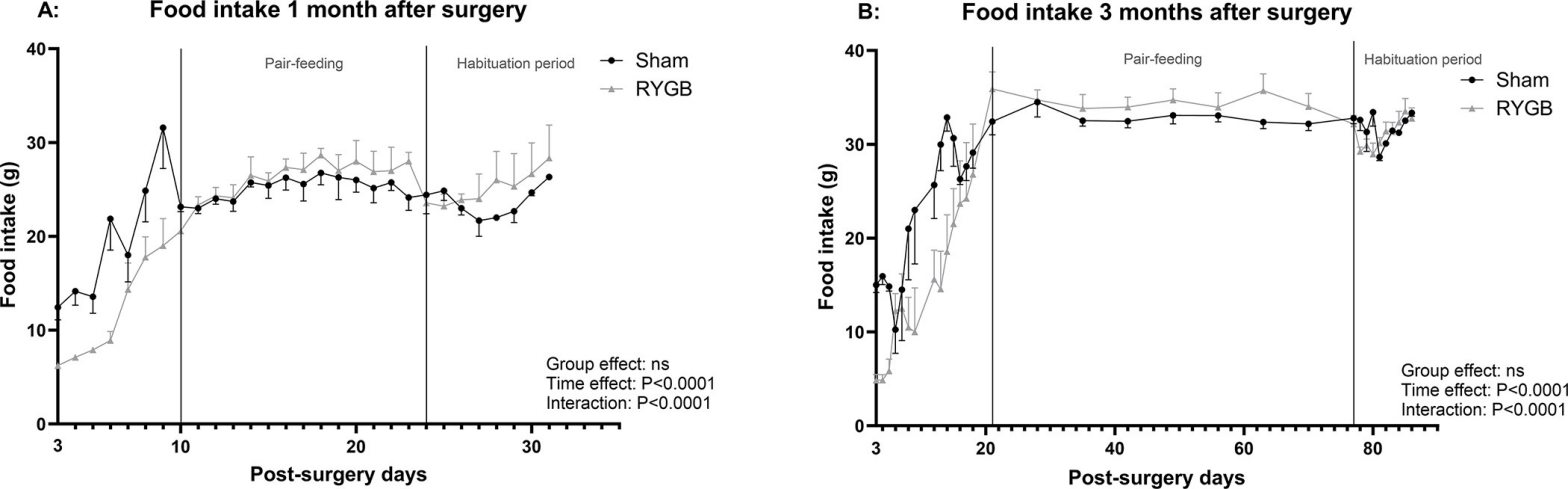
Food intake. Food intake (g) 1 month (A) and 3 months (B) after surgery in Sham and RYGB rats.

**Fig 3:**
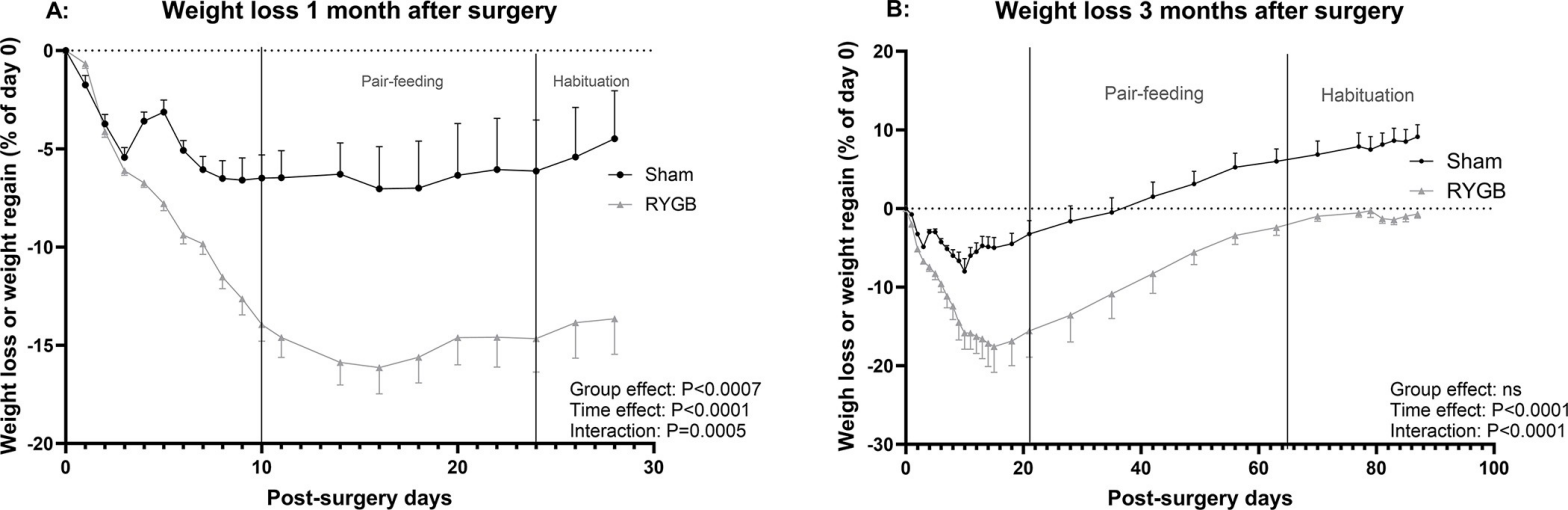
Weight loss. Weight loss (% of initial weight) 1 month (A) and 3 months (B) after surgery in Sham and RYGB rats.

The rats were refed 3 days post-surgery, and pair-feeding started when the food intake was stable. Because the food intake was lower in the RYGB group than in the Sham group (P = 0.009) before pair-feeding, introduction of the latter induced a reduction in food intake among Sham rats. Dietary intake stabilized at around 30g/day (Fig 2). Ten days before the postprandial test, the feeding protocol changed in order to train the rats to eat an entire test meal rapidly in the morning. Food intake decreased in both groups but then increased again to reach the same level as before habituation. Two weeks after surgery, the RYGB rats followed for 1 month had lost around 16±1.5% of their preoperative weight, in contrast to around 6±1.6% among Sham rats (Fig 3). The same trend was observed in the rats followed for 3 months: 2 weeks after the procedure, RYGB rats had lost 17±3.2% of their preoperative weight, as opposed to 5±1.2 for Sham rats. All rats then regained weight until the end of the study, although the RYGB rats regained less than the Sham rats. Three months after surgery, RYGB rats had the same weight as before surgery, while Sham rats regained around 9% more than their initial weight.

### Dietary protein bioavailability

#### N digestibility

Dietary N digestibility was determined by measuring the percentage of N recovered in the lumen of the different gastrointestinal tract (GIT) segments 6h after the meal (Table 1). In the stomach, less dietary N was recovered in RYGB rats than in Sham rats at both time points after surgery (P = 0.03). In the other GIT segments, dietary N recovery was similar in all groups, regardless of the follow-up period. Consequently, there were no differences between dietary N digestibility, around 94.2±0.3% in RYGB and Sham rats.

**Table 1:** Dietary nitrogen recovered in digestive contents after the meal test ingestion in rats followed up for 1 or 3 months after surgery.

% of N ingested							
	1 month		3 months		P value		
	Sham	RYGB	Sham	RYGB	Group	Time	Group*Time
Stomach	0.95±0.29	0.34±0.09	0.98±0.40	0.47±0.24	0.007	ns	ns
Proximal intestine	0.79±0.11	0.86±0.10	0.82±0.21	0.80±0.13	ns	ns	ns
Ileum	0.36±0.14	0.37±0.10	0.72±0.20	0.28±0.06	ns	ns	ns
Cecum	4.32±0.29	4.28±0.36	4.47±0.45	4.18±0.11	ns	ns	ns
Colon + feces	1.53±0.27	1.68±0.50	1.49±0.31	1.55±0.35	ns	ns	ns
**Digestibility**	**93.79±0.26**	**94.42±0.34**	**94.08±1.02**	**94.67±0.24**	ns	ns	ns

n = 8 for Sham followed for 1 month, n = 9 for RYGB rats followed for 1 month, n = 8 for Sham followed for 3 months, n = 7 for RYGB followed for 3 months. Group and time effects were tested using a two-way ANOVA with group and time (1 vs. 3 months) as factors and post hoc Bonferroni tests for pairwise comparisons. Values are means ± SEM. RYGB: Roux-en-Y Gastric Bypass, ns: not significant

### Individual AA digestibility

True cecal AA digestibility was determined by measuring the percentage of ^15^N in AAs recovered from the cecal contents 6h after the meal (S3 Table). Mean cecal AA digestibility was similar in all groups (between 95.5±0.1% and 96.3±0.1%), except for lysine, for which digestibility was slightly but significantly higher after a gastric bypass at both time points during postoperative follow-up (group effect: P = 0.047). The lowest digestibility was for serine (between 88.3±0.7% and 91.1±1.2%, depending on the group) and the highest for phenylalanine (between 98.7±0.1% and 98.9±0.2%).

### Intestinal morphometry

**Fig 4:**
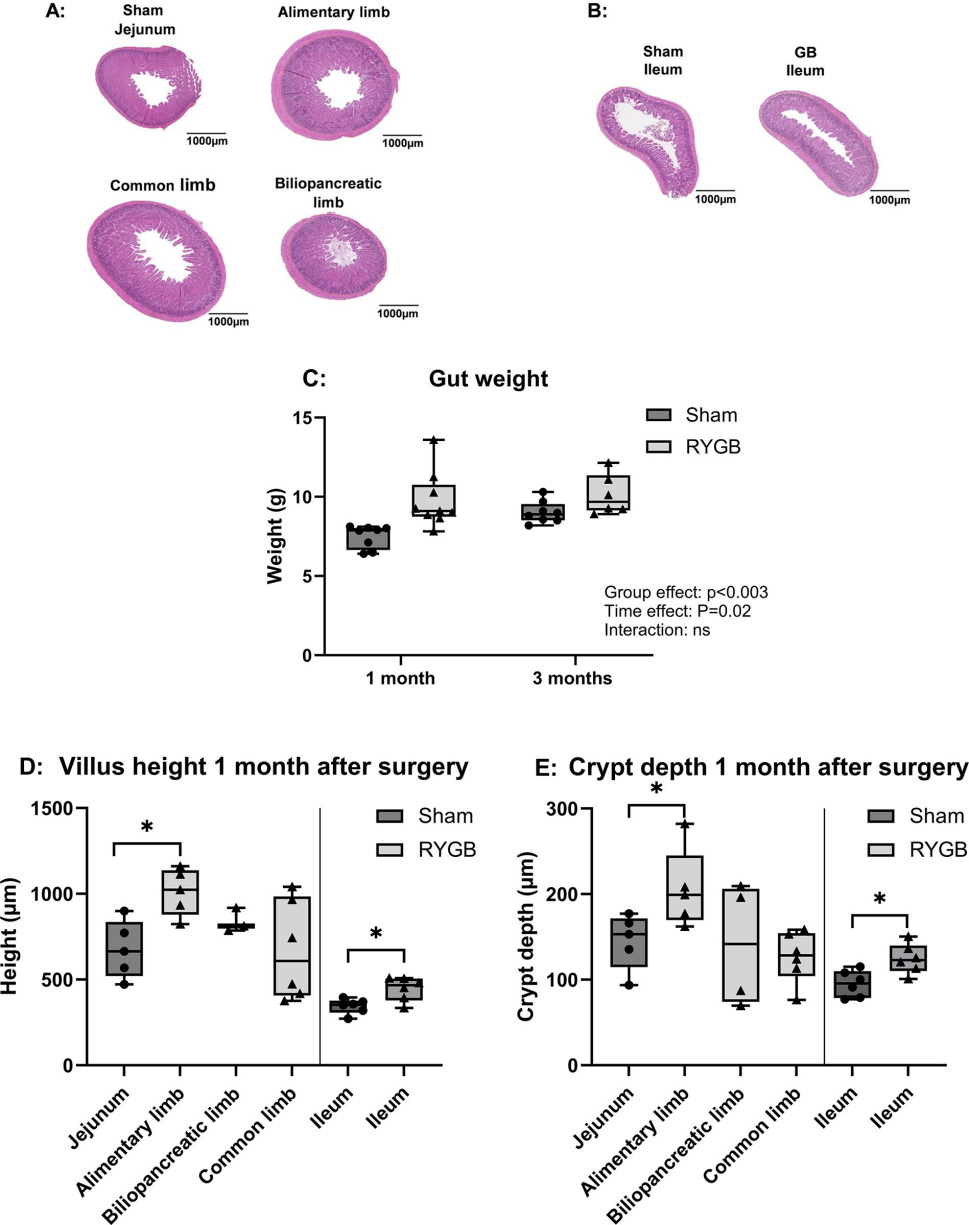
Morphometry of the small intestine. Representative images of the proximal intestine, i.e. the alimentary limb, biliopancreatic limb, and common limb in RYGB rats and the jejunum of Sham rats (A), the ileum of both RYGB and Sham rats (B), and boxplot of small intestine weight (proximal intestine and ileum) after 1 or 3 months post-surgery (C), villus height after 1 month (D), and crypt depth after 1 month (E). The villus heights and crypt depths of the alimentary limb, biliopancreatic limb and common limb of RYGB rats were compared to the Sham jejunum, and the villus heights and crypt depths of RYGB rats ileum were compared to the Sham ileum. n = 8 for Sham followed for 1 month, n = 9 for RYGB rats followed for 1 months, n = 8 for Sham followed for 3 months, n = 7 for RYGB followed for 3 months. The number of data is variable for the villus height and crypt depth because of technical issues. To determine gut-weight differences, group and time effects were tested using a two-way ANOVA with group and time (1 vs. 3 months) as factors and post hoc Bonferroni tests for pairwise comparisons. For differences in villus height and crypt depth, a Student’s t-test was used. Considering the number of tests for villus height and crypt depth (n = 4), a significant difference was determined at 0.05/4 = 0.0125 (Bonferroni correction). *P <0.05. ns: not significant. RYGB: Roux-en-Y Gastric Bypass.

Histological sections of the intestine were collected to analyze the morphometry of the small intestine. The mucosa of the intestinal limbs in RYGB rats was hypertrophied compared to the jejunum mucosa of Sham rats (Fig 4A). The same effect was observed in the ileal mucosa of RYGB rats versus the ileum of Sham rats (Fig 4B).

Accordingly, the weight of the small intestine was higher in RYGB rats than in Sham rats 1 month after surgery (9.1±0.2 vs 7.0±0.3 respectively, P = 0.003), but this effect had disappeared at 3 months (Fig 4C). One month after surgery, villus height in the alimentary limb, but not the biliopancreatic and common limbs, was higher than in the jejunum of Sham rats (P = 0.04). Villus height in the ileum was also higher in RYGB than in Sham rats (P = 0.009) (Fig 4D). These effects had also disappeared 3 months after surgery (S1 Fig). Crypt depth (Fig 4E) was greater in the alimentary limb and ileum of RYGB rats than in the jejunum and ileum of Sham rats 1 month after surgery (P = 0.03 and P = 0.02 respectively). Still, it was similar in the two groups 3 months after surgery (S1 Fig).

### N sequestration in tissues

To evaluate the metabolic bioavailability of dietary N after RYGB, ^15^N retention in GIT tissues, other organs, and blood was assessed. Organ weight was similar between RYGB and Sham, except for the liver, which was lower in RYGB at 3 months, an effect that disappeared after adjustment to body weight (S2 Fig).

### GIT tissues

**Fig 5:**
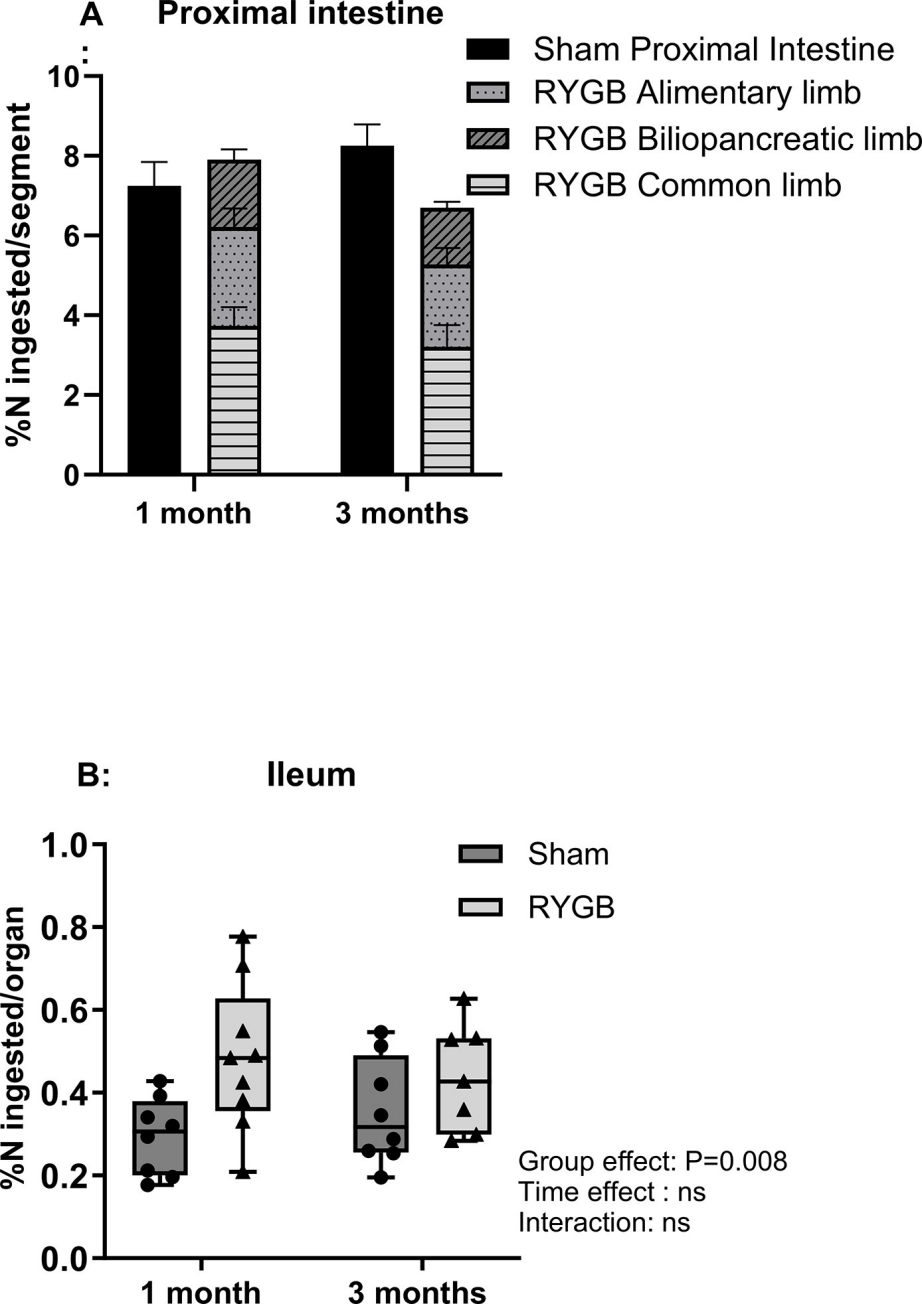
Dietary N recovered from GIT tissues. Dietary N recovered Proximal intestine (duodenum + jejunum) (A) and Ileum (B), expressed as a percentage of N ingested per intestinal segment. n = 8 for Sham followed for 1 month, n = 9 for RYGB rats followed for 1 months, n = 8 for Sham followed for 3 months, n = 7 for RYGB followed for 3 months. Group and time effects were tested using a two-way ANOVA with group and time (1 vs. 3 months) as factors and post hoc Bonferroni tests for pairwise comparisons. *P <0.05. In the column bar graph, values are means ± sem. ns: not significant. RYGB: Roux-en-Y Gastric Bypass.

In gastrointestinal tissues, dietary N retention differed depending on the segments. In the proximal intestine, the proportion of dietary N sequestration was similar between Sham and RYGB rats at both time points after surgery (Fig 5A). In the ileal mucosa, the proportion of dietary N sequestered was higher in RYGB rats than in Sham rats (respectively 0.48±0.18% vs 0.30±0.09% of N ingested, P = 0.04) 1 month after surgery, an effect that tended to persist at 3 months (Fig 5B). There were no significant effects of group, time, and interactions on the proportion of dietary N sequestration in the stomach, cecum, and colon walls.

### Other organs

**Fig 6:**
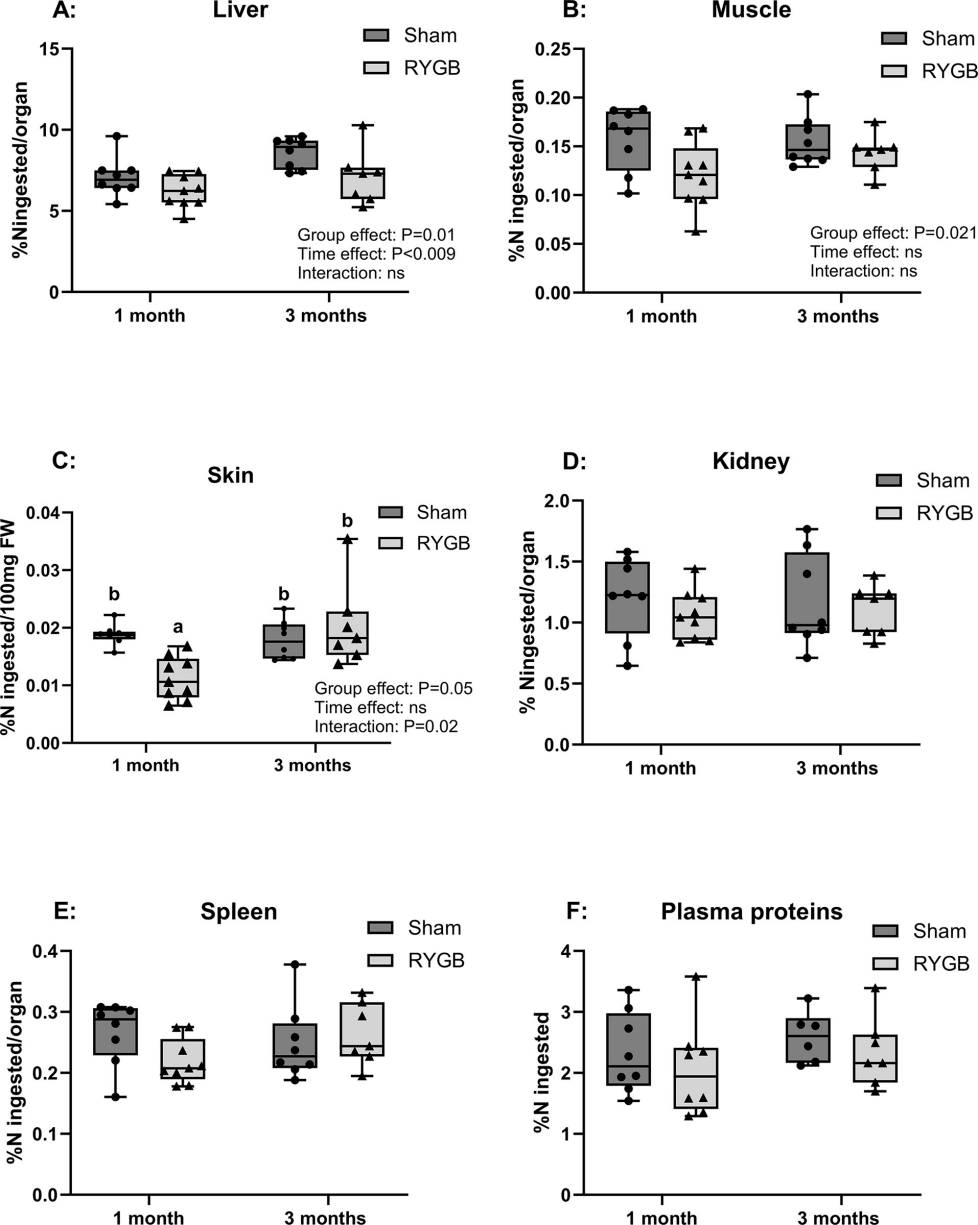
Dietary N recovered from other organs. Dietary N sequestration in the Liver (A), Muscle (B), Skin (C), Kidney (D), Spleen (E), and plasma proteins (F), expressed as a percentage of N ingested per organ or 100mg of Fresh Weight (skin). n = 8 for Sham followed for 1 month, n = 9 for RYGB rats followed for 1 months, n = 8 for Sham followed for 3 months, n = 7 for RYGB followed for 3 months. Group and time effects were tested using a two-way ANOVA with group and time (1 vs. 3 months) as factors and post hoc Bonferroni tests for pairwise comparisons. Values with different letters are statistically different. FW: Fresh Weight, ns: not significant. RYGB: Roux-en-Y Gastric Bypass.

The percentage of dietary N retention in the liver was significantly lower in RYGB rats than in Sham rats (6.2±0.3% VS 7.1±0.4%/liver, P = 0.048) 1 month after surgery, an effect that persisted after 3 months (Fig 6A). In muscle, there was an effect of the group (P = 0.02) on ^15^N retention, which was lower in RYGB (0.12±0.01%/muscle) than in Sham rats (0.16±0.01%) 1 month after surgery, although this difference was attenuated 3 months after surgery (Fig 6B). The same effect was observed in the skin, with a significantly lower recovery in RYGB rats than in Sham rats (0.024±0.003% VS 0.044±0.004%/100mg of fresh weight, respectively, P = 0.021) 1 month after RYGB, but this effect had disappeared 3 months after surgery (Fig 6C). In the kidney, spleen, and blood, the proportion of dietary N retained was similar in both groups, regardless of the duration of follow-up (Fig 6D–6F).

### Fractional synthesis rate

**Fig 7:**
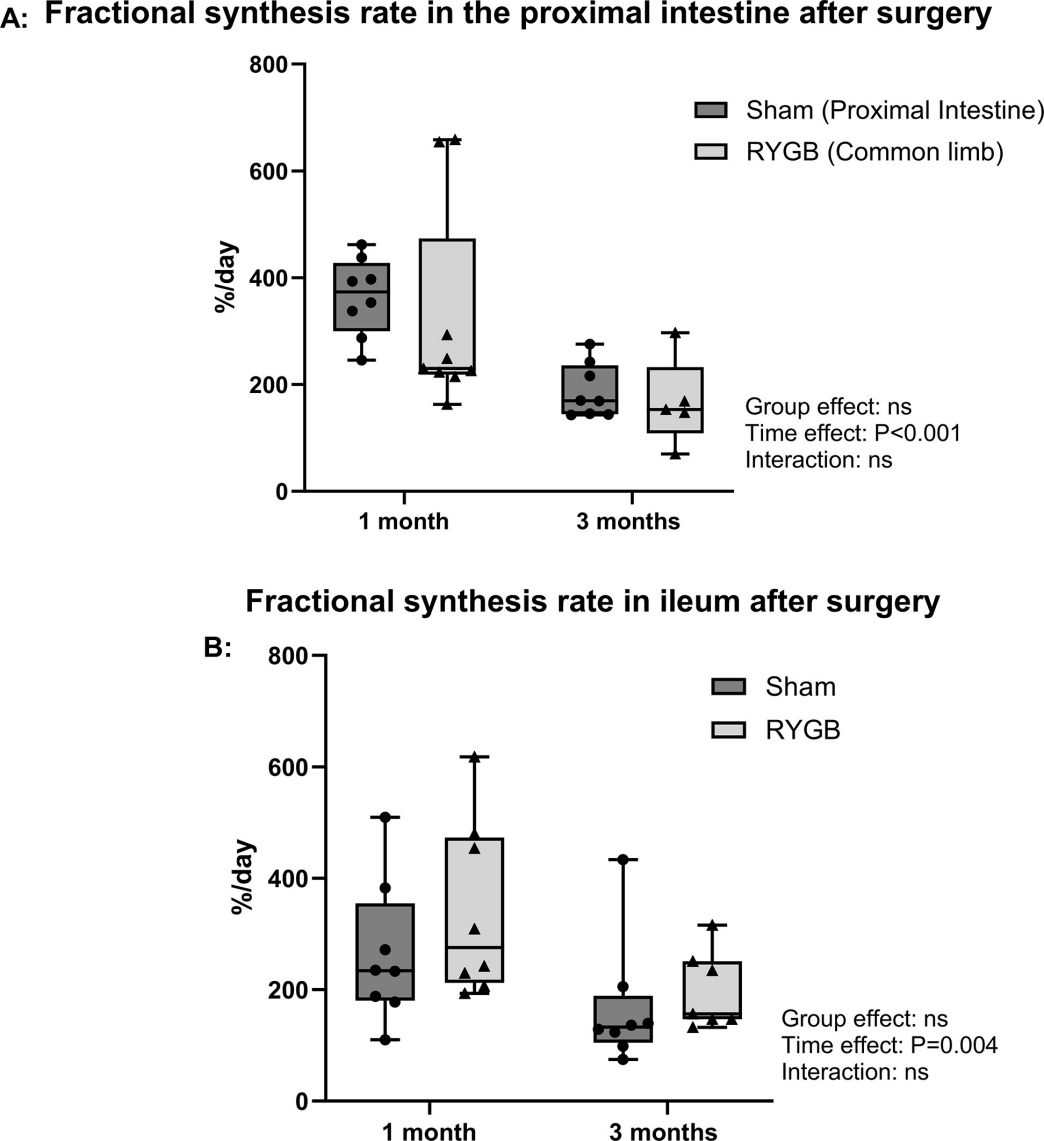
FSR in the proximal intestine and ileum. Fractional synthesis rate in the proximal intestine (A) and ileum (B). n = 8 for Sham followed for 1 month, n = 9 for RYGB rats followed for 1 months, n = 8 for Sham followed for 3 months, n = 7 for RYGB followed for 3 months. Group and time effects were tested using a two-way ANOVA with group and time (1 vs. 3 months) as factors and post hoc Bonferroni tests for pairwise comparisons. The whiskers include both the smallest value and the largest. ns: not significant. RYGB: Roux-en-Y Gastric Bypass.

The fractional synthesis rate of the jejunum and ileal mucosa was higher in rats 3 months after surgery than in those followed for 1 month (time effect: P = 0.022 and P = 0.004, respectively), but no significant group effect was observed (Fig 7).

### Metabolic losses

To assess the dietary N lost through deamination, ^15^N was determined in the urines and plasma urea (Table 2). The percentage of ingested N lost in urine was higher in RYGB than in Sham rats at 1 month (an absolute increase of 2.9%), an effect that diminished after 3 months (RYGB vs Sham, P = 0.07). The same effect was observed for ^15^N recovered in plasma urea. Consequently, there was a group effect regarding total deamination, which was higher in RYGB rats than in Sham rats, regardless of the follow-up period.

**Table 2:** Dietary N recovered from metabolic losses 1 or 3 months after surgery.

% N ingested							
	1 month		3 months		P value		
	Sham	RYGB	Sham	RYGB	Group	Time	Group*Time
Urinary urea	7.0±0.5	9.9±0.9	6.4±0.8	8.2±1.3	P = 0.014	ns	ns
Plasma urea	2.9±0.2	4.2±0.3	3.2±0.2	4.3±0.2	P = 0.0001	ns	ns
Total deamination	10.0±0.7	14.0±1.2	9.6±0.8	12.4±1.22	P = 0.003	ns	ns

n = 8 for Sham followed for 1 month, n = 9 for RYGB rats followed for 1 month, n = 8 for Sham followed for 3 months, n = 7 for RYGB followed for 3 months. Group and time effects were tested using a two-way ANOVA with group and time (1 vs. 3 months) as factors and post hoc Bonferroni tests for pairwise comparisons. Values are means ± SEM. RYGB: Roux-en-Y Gastric Bypass, ns: not significant

## Discussion

This study assessed the time course (1 to 3 months) effects of gastric bypass on intestinal adaptations and the postprandial handling of dietary protein after a single test meal, including ^15^N-labeled protein. The study showed that RYGB did not alter protein digestibility and induced intestinal mucosa hypertrophy. However, a higher sequestration of dietary N was observed only in the ileum. By contrast, there was a temporary and moderate decrease in dietary N recovery from several organs, which was associated with a marked and sustained increase in postprandial deamination after RYGB.

At the beginning of the study, the rats were 24 weeks old [40] what corresponds to approximately 25–30 years in humans [24]. The rats were followed during 1 month or 3 months after surgery, corresponding to around 1,5 years and 5 years, respectively, in humans. After surgery, Sham rats were pair-fed with RYGB rats once dietary intake had stabilized. RYGB rats lost more weight than Sham rats. Weight regain was seen in all groups around two weeks after surgery, according to the continuous growth during the adult life of rats. It can be noticed that around one month after surgery, the weight of Sham rats was higher than before surgery, which is due to a higher caloric intake. Such a weight regain has also been observed at 40 days by Shin et al. [41]. Overall, the weight of RYGB rats was lower than that of Sham rats despite pair-feeding. Food intake remained at the same level as before surgery in all group rats, which was inconsistent with another study showing that RYGB induced weight loss independently of food intake [42].

Dietary N bioavailability was assessed using a ^15^N test meal to determine protein digestibility, ^15^N retention in organs, and losses through urea. This test had previously made it possible to reveal protein malabsorption in the context of pancreatitis in humans and minipigs [43, 44]. Protein digestibility was around 94%, consistent with many studies on casein digestibility in rats, pigs, and humans [27, 45–48]. This value was similar in RYGB and Sham rats at both time points after postoperative recovery. These findings differ from our previous study, indicating that protein digestibility was slightly and paradoxically higher in RYGB rats [20]. This difference may be ascribed to the observation time after surgery, i.e., 3 weeks instead of 1 month in the present study. Protein digestibility may temporarily increase in the short term (<1 month) after RYGB due to intense intestinal remodeling. Nevertheless, the effects of RYGB on protein malabsorption are still debated. Reducing the production of gastric hormones such as gastrin can decrease the secretion of pancreatic enzymes [49], although total gastrectomy did not induce protein malabsorption in patients [50]. In a study based on five RYGB patients, the arrival of biliopancreatic secretions (including trypsin) in the small intestine was delayed, resulting in protein malabsorption [51]. Among trials that have evaluated protein digestion and absorption in patients after RYGB, some reported protein malabsorption [17], and others revealed inter-individual variability in the effect [16]. The accelerated appearance of dietary AAs was also reported after RYGB [18, 19], but this might solely be due to the more rapid gastric emptying induced by this surgery [52].

As expected, analysis of the small intestine anatomy revealed hypertrophy after RYGB. One month after surgery, the intestinal weight was higher in RYGB than in Sham rats, but no differences were observed at 3 months. Interestingly, the intestinal weight of Sham rats increased between 1 and 3 months, in contrast to the RYGB animals. As the Sham rats gained more weight than RYGB, adjusting intestinal weight to total body weight revealed a significant difference between the groups. Higher villus height and deeper crypts after RYGB were also observed, particularly in the alimentary limb and ileum one month after surgery. However, this effect disappeared at three months. Such hypertrophy has been observed in rats in the short, medium [20, 23, 25], and long-term after surgery, including ten months [21]. However, the latter study used a mix of standard and high-fat diets throughout the experiment, unlike our study, where rats were refed a standard diet. Intestinal hypertrophy has been suggested as an adaptation to RYGB that leads to modified hormonal profiles and glucose metabolism [22]. Increased glucose uptake in the small intestine, with higher sequestration and metabolization of glucose, has been reported and proposed as a mechanism contributing to the observed decrease in glycemia after RYGB [25]. Consequently, intestinal hypertrophy may increase intestinal protein requirements, resulting in the sequestration of dietary protein by the small intestinal mucosa at the expense of other organs. However, our results indicated no difference in dietary N sequestration, except in the ileum, where it was higher in RYGB rats when expressed both per unit of tissue and per total tissue weight. In other segments, there were no significant differences in ^15^N recovery when adjusted for tissue weight.

Intestinal cell turnover may be accelerated after RYGB. In a study on intestinal adaptation after jejunoileal bypass in 12 men, the thickness of the intestinal mucosa was not altered, but crypt depth increased [53]. Another trial involving 19 RYGB patients who were followed for 6 months suggested an increase in intestinal cell turnover based on a stronger expression of markers for intestinal stem cells, Paneth cells, and goblet cells, while enterocyte markers were downregulated [54]. However, our findings regarding the small intestine FSR do not support this hypothesis, as there were no significant differences between the groups. Interestingly, FSR decreased significantly in all segments from 1 to 3 months, which may have been associated with growth deceleration. The same effect had already been described in another study where the muscle FSR was lower in older rats than in young rats, although the FSR in these two organs is not directly comparable. To our knowledge, no data are available regarding the time course of intestinal wall FSR during aging.

Dietary N sequestration was evaluated in other organs and was lower in the liver, muscle, and skin in RYGB compared to Sham rats at 1 month after surgery. This effect was of moderate extent at this point and disappeared after 3 months. A similar trend was observed in the kidney, spleen, and plasma proteins. When adjusted for tissue weight, ^15^N retention in all tissues was also lower after RYGB (S3 Fig), indicating that this effect was not solely due to a confounding factor related to organ weight. These results are consistent with those reported in our previous study [20]. In the absence of intestinal malabsorption, this decrease in N retention in organs could be explained by increased metabolic losses induced by RYGB. Interestingly, our study confirmed this hypothesis as ^15^N recovery in both plasma and urinary urea was higher after RYGB, resulting in a 4% increase in total deamination compared to Sham rats. This rise in deamination after surgery could partly be attributed to the faster AA absorption mentioned earlier [18, 19]. The rapid appearance of AAs has been shown to increase dietary N loss through the urea cycle [27]. Further, it can be hypothesized that protein turnover is enhanced by the significant weight loss observed following RYGB. Approximately 30% of lean mass loss has been attributed to weight loss in patients 12 months after RYGB surgery [55, 56], which may increase protein and AA catabolism.

Most of the effects observed during our study, except for deamination, were transient, particularly regarding dietary N sequestration in tissues. Clinical studies have also reported transient effects, such as decreased levels of albumin and pre-albumin in the short term after RYGB, which do not persist over time [8, 57]. These findings suggest several adaptive mechanisms were initiated after RYGB gradually disappeared, facilitating a new balance for the organism.

This study had some limitations. Firstly, the relatively small number of animals in each group was due to mortality following the RYGB procedure. Secondly, we compared non-anatomically equivalent intestinal segments, i.e., the three limbs of RYGB rats and the middle section of the jejunum of Sham rats, because the three limbs had initially formed part of the jejunum. However, the relationship between these anatomical segments is a subject of debate, and it would have been interesting to sample the proximal, middle, and distal jejunum in Sham rats. Lastly, the pair-feeding of Sham rats with RYGB rats during this study was necessary to avoid any confounding effect of food intake, but this resulted in caloric restriction in Sham rats, which could alter protein metabolism. Comparing these data to Sham rats not subjected to caloric restriction might have been informative. Lastly, our ^15^N meal approach has not been implemented in bariatric surgery patients, making our data hardly generalizable to humans.

In conclusion, this study provides unique data on quantifying dietary protein distribution, including metabolic losses, at different times after RYGB. The results confirmed that RYGB does not affect protein digestibility but induces hypertrophy of the intestinal mucosa. However, an increase in dietary N sequestration was only observed in the ileum. There was a moderate and transitory reduction in dietary N retention from other organs but a marked and sustained increase in postprandial deamination after RYGB, which warrants further investigation. Except for deamination, these effects appeared transient and disappeared once the organism reached a new balance post-surgery. Those findings warrant to be confirmed in humans. If the efficiency of dietary proteins is diminished after gastric bypass surgery, it may be advisable for patients undergoing RYGB to consider a higher protein intake than the currently recommended 60g/day.

## Supporting information

S1 Fig Villus height and crypt depth 3 months after surgery. Villus height (A) and crypt depth (B) in the small intestine.
(TIF)

S2 Fig Organ weight. Organ weight (A) and organ weight reported per g body weight.
(TIF)

S3 Fig Dietary N recovered in other organs. Dietary N sequestration was assessed in the Liver (A), Muscle (B), Kidney (C), Spleen (D), expressed in % of N ingested per 100mg of Fresh Weigh.
(TIF)

S1 Table Composition High Fat Diet (TD.08811). Composition in mass (g/kg of dry matter (DM)) and energy supply (kcal/kg).
(DOCX)

S2 Table Composition standard diet. Composition in mass (g/kg of dry matter (DM)) and energy supply (kcal/kg).
(DOCX)

S3 Table Composition standard diet. True cecal amino acid digestibility (%) measured in rats followed for 1 or 3 months after surgery.
(DOCX)

## Abbreviations

AA: amino acid
FSR: fractional synthesis rate; Gastrointestinal Tract
GIT: GC, gas chromatography
HFD: high fat diet
IRMS: isotopic ratio mass spectrometry
MS: mass spectrometry
N: nitrogen
RFD: real fecal digestibility
RYGB: Roux-en-Y gastric bypass
UHPLC: Ultra High-Performance Liquid Chromatography
