# Supplementary figures and images for: Effects of gastric bypass on the digestibility and postprandial metabolic fate of 15N dietary protein in rats
Source: PLoS One. 2024 Aug 5;19(8):e0307075. doi: 10.1371/journal.pone.0307075 (PMC11299818; doi:10.1371/journal.pone.0307075)

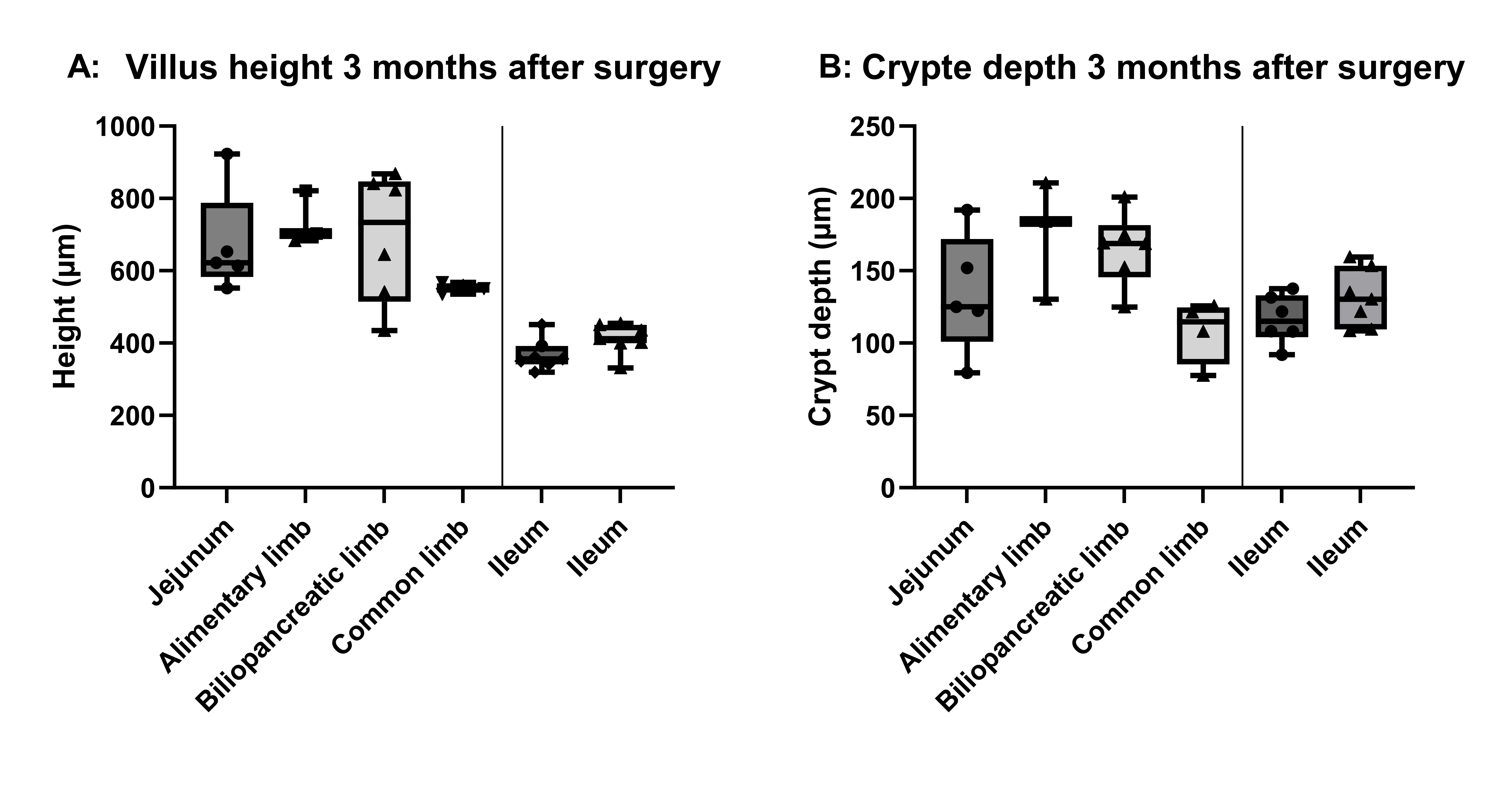

Supplement: S1 Fig — Villus height (A) and crypt depth (B) in the small intestine. (TIF) [file pone.0307075.s001.tif]

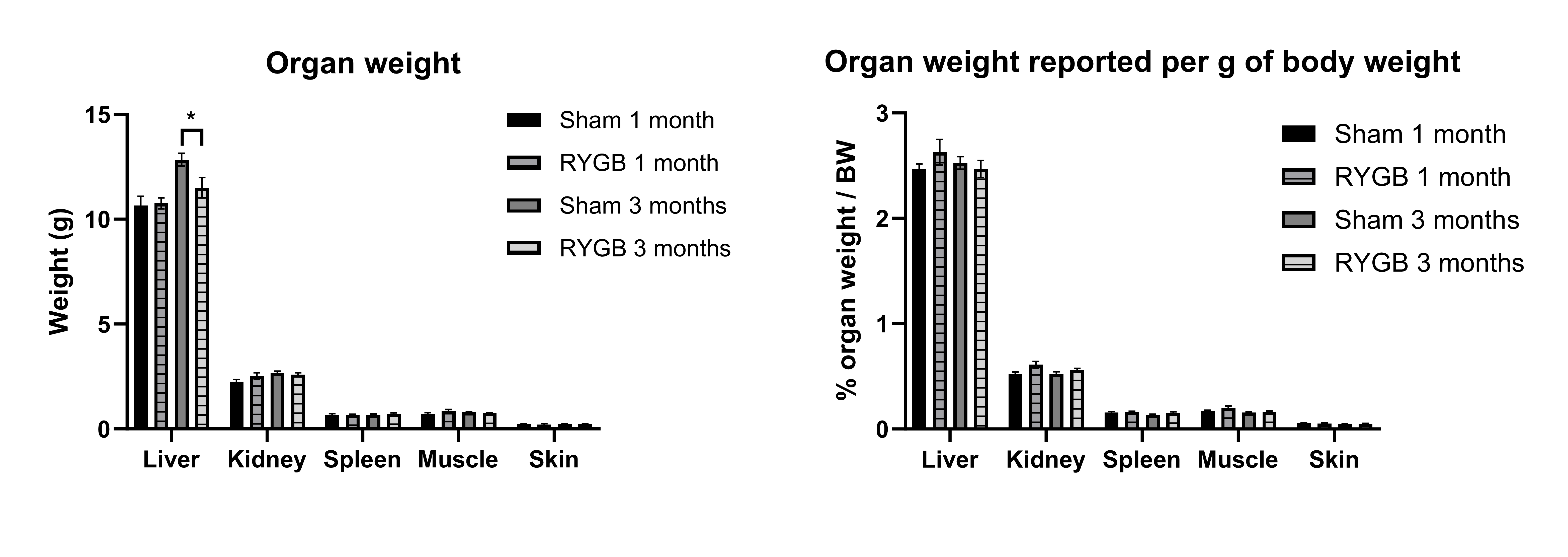

Supplement: S2 Fig — Organ weight (A) and organ weight reported per g body weight. (TIF) [file pone.0307075.s002.tif]

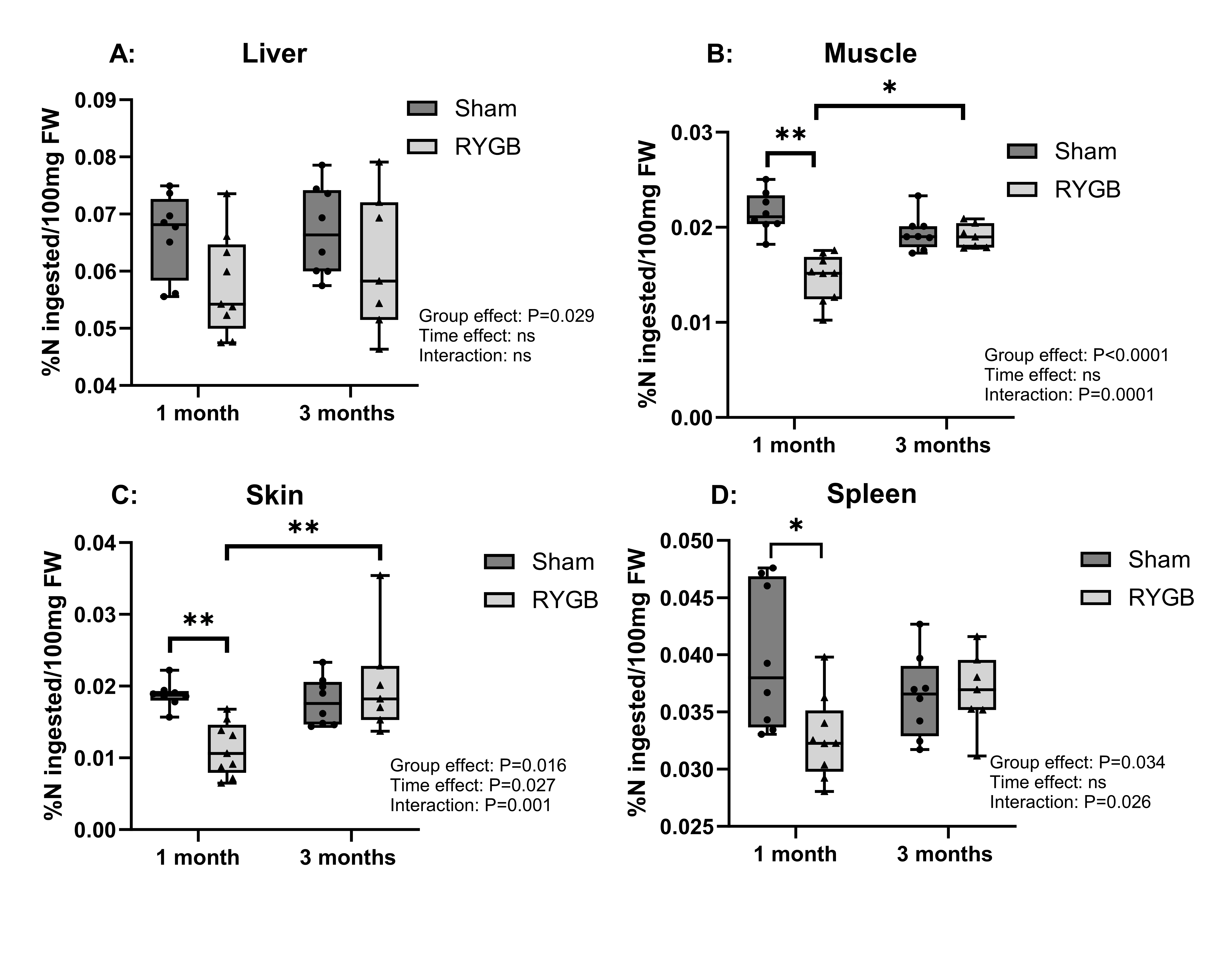

Supplement: S3 Fig — Dietary N sequestration was assessed in the Liver (A), Muscle (B), Kidney (C), Spleen (D), expressed in % of N ingested per 100mg of Fresh Weigh. (TIF) [file pone.0307075.s003.tif]
