# Supplementary material for: Effects of gastric bypass on the digestibility and postprandial metabolic fate of 15N dietary protein in rats
Source: PLoS One. 2024 Aug 5;19(8):e0307075. doi: 10.1371/journal.pone.0307075 (PMC11299818; doi:10.1371/journal.pone.0307075)
Supplement: S1 Table — Composition in mass (g/kg of dry matter (DM)) and energy supply (kcal/kg). (DOCX) [file pone.0307075.s004.docx]

**Table S1. Composition High Fat Diet (TD.08811).**

| HFD (TD.08811)  45%kcal Fat Diet (21% MF, 2% SBO) | |
| --- | --- |
|  | g/kg DM |
| Casein | 195 |
| L-Cystine | 3 |
| Sucrose | 340 |
| Corn starch | 56.86 |
| Maltodextrin | 60 |
| Anhydrous Milkfat | 210 |
| Soybean oil | 20 |
| Cellulose | 50 |
| Minerals^1^ | 43 |
| Vitamins^1^ | 19 |
| Choline Bitartrate | 3 |
| TBHQ, antioxidant | 0.04 |
| Green Food Color | 0.1 |

Compositions are expressed in g/kg of dry matter (DM).

^1^Minerals and vitamins were formulated from AIN-93M.
