# Supplementary material for: Effects of gastric bypass on the digestibility and postprandial metabolic fate of 15N dietary protein in rats
Source: PLoS One. 2024 Aug 5;19(8):e0307075. doi: 10.1371/journal.pone.0307075 (PMC11299818; doi:10.1371/journal.pone.0307075)
Supplement: S2 Table — Composition in mass (g/kg of dry matter (DM)) and energy supply (kcal/kg). (DOCX) [file pone.0307075.s005.docx]

**S2 Table. Composition standard diet.**

| Standard diet P14 | |
| --- | --- |
|  | g/kg DM |
| Protein | 120 |
| Soybean oil | 42 |
| Starch | 630 |
| Sucrose | 110 |
| Cellulose | 50 |
| Minerals^1^ | 35 |
| Vitamins^1^ | 10 |
| Choline | 2.3 |

Compositions are expressed in g/kg of dry matter (DM).

^1^Minerals and vitamins were formulated from AIN-93M.
