# Supplementary material for: Effects of gastric bypass on the digestibility and postprandial metabolic fate of 15N dietary protein in rats
Source: PLoS One. 2024 Aug 5;19(8):e0307075. doi: 10.1371/journal.pone.0307075 (PMC11299818; doi:10.1371/journal.pone.0307075)
Supplement: S3 Table — True cecal amino acid digestibility (%) measured in rats followed for 1 or 3 months after surgery. (DOCX) [file pone.0307075.s006.docx]

**S3 Table. True cecal amino acid digestibility (%) measured in rats followed for 1 or 3 months after surgery.**

|  | **1 month** | | **3 months** | | **p-value** |
| --- | --- | --- | --- | --- | --- |
|  | **Sham** | **RYGB** | **Sham** | **RYGB** | **Group effect**  **(p-value)** |
| **Alanine** | 95.06±0.50 | 94.02±0.30 | 95.10±0.82 | 95.01±0.64 | ns |
| **Glutamate** | 94.12±0.37 | 95.27±0.31 | 94.46±1.24 | 96.12±0.80 | ns |
| **Glycine** | 94.49±0.51 | 93.65±0.34 | 93.82±0.95 | 94.78±0.63 | ns |
| **Isoleucine** | 93.37±0.58 | 93.94±0.31 | 93.74±0.66 | 94.24±0.35 | ns |
| **Leucine** | 96.21±0.32 | 96.70±0.16 | 96.57±0.58 | 97.09±0.26 | ns |
| **Lysine** | 97.76±0.28 | 97.98±0.11 | 97.50±0.20 | 98.12±0.05 | 0.046 |
| **Methionine** | 95.19±0.34 | 95.57±0.34 | 95.84±0.75 | 96.08±0.47 | ns |
| **Phenylalanine** | 98.93±14 | 98.69±0.08 | 98.98±0.15 | 98.93±0.08 | ns |
| **Proline** | 97.78±0.14 | 97.92±0.10 | 97.71±0.46 | 98.17±0.22 | ns |
| **Serine** | 88.27±0.72 | 90.21±0.58 | 88.81±2.26 | 91.13±1.21 | ns |
| **Threonine** | 96.09±0.34 | 96.29±0.22 | 96.95±1.09 | 97.63±0.64 | ns |
| **Tyrosine** | 98.92±0.15 | 98.61±0.07 | 98.85±0.20 | 98.78±0.09 | ns |
| **Valine** | 94.79±0.41 | 95.09±0.23 | 94.75±0.56 | 95.21±0.18 | ns |
